# Characteristics of high frame frequency contrast-enhanced ultrasound in renal tumors

**DOI:** 10.1186/s12880-024-01245-0

**Published:** 2024-03-25

**Authors:** WeiPing Zhang, JingLing Wang, Li Chen

**Affiliations:** Department of Ultrasound, The First Affiliated Hospital, Jiangxi Medical College, Nanchang University, Nanchang, China

**Keywords:** High frame frequency, Contrast-enhanced ultrasound, Renal tumors, Characteristics, Analysis

## Abstract

**Objective:**

This study aims to analyze the characteristics of high frame rate contrast-enhanced ultrasound (H-CEUS) in renal lesions and to improve the ability for differential diagnosis of renal tumors.

**Methods:**

A total of 140 patients with renal lesions underwent contrast-enhanced ultrasound (CEUS) examination in the First Affiliated Hospital of Nanchang University from July 2022 to July 2023. Based on the tumor pathology and the results of enhanced CT, tumor patients were divided into malignant and benign groups. All subjects were examined using gray-scale ultrasound, conventional contrast-enhanced ultrasound (C-CEUS), and H-CEUS, and their dynamic images were recorded. Two radiologists independently analyzed and recorded the results of ultrasound, C-CEUS, and H-CEUS images and statistically analyzed the features of C-CEUS and H-CEUS images. The independent sample *t*-test was used to compare the difference in age and maximum diameter of nodules between the benign and malignant groups. The *χ*^*2*^ test was used to compare the sex, mode of operation, gray-scale ultrasound characteristics, and enhancement characteristics of the two CEUS modes (enhancement mode, regression mode, enhancement degree, enhancement uniformity, enhancement or not, enhancement direction, post-enhancement boundary and range, and pseudocapsule) between the benign and malignant groups. The difference in vascular morphology of malignant nodules of varying sizes under two angiographic modes.

**Results:**

There were significant differences in gender (*χ*^*2*^ = 10.408, *P* = 0.001), mode of operation (*χ*^*2*^ = 47.089, *P* < 0.001), nodule composition (*χ*^*2*^ = 7.481, *P* = 0.003), nodule echo (*χ*^*2*^ = 20.926, *P* < 0.001), necrosis (*χ*^*2*^ = 31.343, *P* < 0.001) and nodule blood flow (*χ*^*2*^ = 9.006, *P* = 0.029) between the benign and malignant groups. There were significant differences in the regression model (*χ*^*2*^ = 6.782, *P* = 0.034) and enhancement direction (*χ*^*2*^ = 13.771, *P* = 0.001) between the two radiographic techniques in the malignant group. There was a significant difference in the enhancement uniformity between the two CEUS techniques in the benign group (*χ*^*2*^ = 8.264, *P* = 0.004). There was a significant difference between the two CEUS techniques in displaying the vascular morphology in the malignant group with the maximum diameter of nodules ≤ 4.0 cm (*χ*^*2*^ = 11.421, *P* < 0.022). However, there was no significant difference between the two techniques in the malignant group with the maximum diameter of nodules > 4.0 cm.

**Conclusion:**

Increasing the frame rate of ultrasound images is helpful to accurately display the enhanced features and vascular morphology of renal tumors, especially for malignant tumors with a maximum diameter of ≤ 4.0 cm. Thus, H-CEUS can make up for the limitation of CEUS with regard to the display of vascular morphology.

## Introduction

Unexplained kidney mass is a prevalent clinical issue; more than half of patients over 50 years old are estimated to have at least one kidney lesion [1]. Renal lesions include benign and malignant renal tumors. Renal angiomyolipoma (AML) is the most common benign renal tumor. Renal cell carcinoma (RCC) is the most common primary malignant tumor of the kidney, accounting for 90–95% of malignant renal tumors, and often requires surgical resection. Approximately 70% of RCC are clear cell renal cell carcinoma (ccRCC), and other rare types of non-clear cell renal cell carcinoma include papillary renal cell carcinoma (pRCC), chromophobe renal cell carcinoma (chRCC), Xpll.2 Translocation/TFE3 Gene Fusions, and collecting duct carcinoma [2–3]. In addition, cystic renal cell carcinoma (CRCC), which is a rare subtype of renal cell carcinoma accounting for 10-15% of the total number of renal cell carcinoma(RCC) and 1-15% of renal tumors [4], has a low-grade malignancy with low pathological staging characteristics. Research has suggested that the correlation between tumor biology and prognosis of CRCC increases with the increase in tumor size [4]. CRCC is usually difficult to distinguish from complex cysts with similar ultrasonic manifestations, including infectious cysts, hemorrhagic cysts, and simple cysts with septa. The common symptoms of RCC include hematuria, low back pain, and palpable abdominal lumps, but these symptoms occur only in 4% of the cases. The most common distant metastases involve the lungs, bones, and brain. Approximately 20% of RCC patients have early metastases during diagnosis, 30% progress to local metastases, and approximately 40% with localized RCC have distant metastases after surgery [5]. Therefore, early diagnosis of RCC is crucial for facilitating patient treatment and minimizing mortality. Ultrasound—widely available, radiation-free, and relatively inexpensive—is essential in determining the characteristics of focal renal mass. It is used to distinguish benign tumors from solid malignant renal tumors and to evaluate the complexity of renal cystic tumors [6]. Furthermore, the widespread application of contrast-enhanced ultrasound (CEUS) has become more prevalent, enabling researchers to examine the enhanced characteristics of renal lesions [7–8]. CEUS is a method to improve the contrast of echoes between normal tissues and lesions through the high-intensity nonlinear harmonic signals produced by contrast media based on the characteristics of microvascular perfusion in the lesion area. CEUS is an examination method to improve the qualitative diagnosis rate and detection rate of lesions [9]. SonoVue is a pure intravascular ultrasound contrast agent with no nephrotoxicity or hepatotoxicity and will not enter the collecting tube. It is exhaled through the lungs for excretion. Multiple injections are allowed with repeated examinations because of the short half-life of SonoVue (approximately 5 min) [10]. CEUS exhibits a strong background tissue inhibition; thus, it is more sensitive than enhanced CT in showing blood flow in the context of very scanty blood supply, and it can also distinguish between solid oligovascular masses and atypical cystic masses [11]. It is more sensitive to display septum and enhancement of solid components than enhanced CT and MRI because of good background suppression of complex renal masses [12].

CEUS infers the tissue type of tumor using data such as enhancement mode, regression mode, peak intensity, enhancement direction, and enhancement shape. For instance, ccRCC often shows early inhomogeneous enhancement and rapid regression. The enhancement margin around the lesion described as pseudocapsule is usually apparent in the late stage of regression, which is another characteristic manifestation of diagnostic ccRCC [13]. Conversely, pRCC usually shows progressive enhancement and persistent low enhancement [14]. The enhancement degree of AML is lower than that of the adjacent renal cortex [15]. In clinical practice, CEUS is not reliable for tumor differentiation, and the overlap of different tissue types is apparent. It is essentially impossible to reliably distinguish AML, oncocytoma, and malignant tumor [16]. Although CEUS is considered to be a real-time imaging technology, its current limitations stem from the frame rate constraint of angiographic images. When the tumor is small and rich in blood supply, the arterial phase perfusion process of CEUS is extremely rapid, highlighting the constraint of the low frame frequency. Consequently, the perfusion process of the tumor and the morphology of blood vessels are difficult to discern with precision, thereby impeding the diagnostic efficiency.

High frame rate contrast-enhanced ultrasound (H-CEUS) imaging technology generally uses a few emission events to complete the target imaging of the specified area. However, the transmitted ultrasound field often chooses the plane wave sound field, enabling the acquisition of imaging information of a rectangular region. Furthermore, the contrast frame rate is increased to 50–80 Hz, which is significantly higher than that of the current clinical use of CEUS (10–15 Hz). To some extent, the increased contrast frame rate improves the adverse effect of low frame frequency on the time resolution of contrast images. By increasing the frame frequency of the contrast image, its time resolution can be improved, thereby providing more diagnostic information and a clearer and more accurate depiction of the contrast medium perfusion process [17]. The present study retrospectively analyzed the ultrasonographic features of C-CEUS and H-CEUS in 140 cases of renal tumors to improve the ability to differentially diagnose benign and malignant renal tumors and to guide clinical decision-making.

## Methods

### Study population

From July 2022 to July 2023, 140 patients with renal mass were examined using C-CEUS and H-CEUS in the first affiliated Hospital of Nanchang University. Inclusion criteria included the following: (1) Gray-scale ultrasound can clearly show renal masses; (2) all malignant lesions should be confirmed through postoperative pathology; (3) all benign lesions should be confirmed through postoperative pathology or enhanced CT and follow-up for 3–6 months; (4) all patients were examined using C-CEUS and H-CEUS, and the dynamic image time was more than 2 min; (5) age ≥ 18 years old; (6) the diameter of lesions was larger than 1 cm. Exclusion criteria were as follows: (1) patients with other renal diseases; (2) patients with poor storage of dynamic images and patients with large respiratory range, which cannot be analyzed in the later stage; (3) patients with CEUS images that cannot be interpreted because of deep tumor location, obesity, and ultrasonic penetration;(4) patients with solid-cystic malignant lesions diagnosed as Bosniak 2/3 by CEUS. This study included 140 patients; the lesions were divided into benign and malignant groups based on the results of pathology and contrast-enhanced CT, including the malignant group (*n* = 99), the benign group (*n* = 41), male (*n* = 74), and female (*n* = 66). In 41 cases of benign lesions, there were 3 cases of epithelioid AML, 21 cases of other AML (include lipid rich or poor AML), 7 cases of congenital variation, 4 cases of eosinophil tumor, 5 cases of inflammatory nodule, and 1 case of metanephric adenoma. Among the 99 cases of malignant lesions, 69 cases were ccRCC (3 cases with sarcomatoid degeneration), 14 cases were pRCC (8 cases of type I and 6 cases of type II), 4 cases were chRCC, 2 cases were collecting carcinoma, 3 cases were RCC associated with Xp11.2 translocation/TEF3 gene fusion, and 7 cases were unclassified RCC. This study was approved by our hospital’s Medical Ethics Committee (IIT2023174), and informed consent was obtained from every participant.

### Examination technique

The Mindray Resona R9 color Doppler ultrasound diagnostic instrument was used with a probe of SC 5-1U and a frequency of 3–5 MHz. The contrast-enhanced ultrasound technology was ultra-wideband nonlinear imaging technology. The frame rate of C-CEUS imaging was 10 Hz, and the frame rate of H-CEUS was 35–45 Hz. The two contrast methods used a low mechanical index of 0.06–0.08. The ultrasound contrast agent was SonoVue (Bracco company, Italy), and the main component was sulfur hexafluoride microbubbles. Before use, 59 mg of SonoVue was added to 5 mL of 0.9% sodium chloride solution, thoroughly shaken, and mixed to form a suspension.

### Implementation of H-CEUS

Routine ultrasound and CEUS examination of the kidney were performed before the operation or enhanced CT. The location, number, size, echo characteristics, and color blood flow of the lesions were recorded using conventional ultrasound. We selected the long axis section where the lesion and the surrounding normal renal tissue exist concurrently, switched to a CEUS mode, injected 1.0 mL of SonoVue suspension through the superficial vein of the elbow, and then again injected 5 mL of 0.9% sodium chloride solution into the tube. The C-CEUS examination was performed first, followed by the H-CEUS examination with a minimum 10-min interval between the two until the microbubbles had disappeared entirely, and then the H-CEUS examination was performed. The two CEUS kinds continuously recorded and stored the dynamic image for 2–3 min. Without being informed of the pathology or the results of the enhanced CT scan, the images were analyzed by two ultrasound specialists with over 5 years of experience in contrast-enhanced ultrasound.

### Ultrasound image features

The following features of conventional ultrasound were recorded: (1) nodule side (left or right); (2) nodule position (upper, middle, or lower pole); (3) nodule composition (solid or Solid-cystic); (4) nodule echo (hyperechoic, hypoechoic, or isoechoic); (5) nodule boundary (clear or unclear); (6) nodule shape (regular or irregular); (7) necrosis (present or absent); (8) the tumor blood flow signals were observed through color Doppler flow imaging [16]: grade 0 (no blood flow in the tumor), grade I (a small amount of 1–2 star-shaped blood flow), grade II (moderate blood flow showed 3–4 star-shaped or short bundles of blood flow), and grade III (rich blood flow showed 2–3 or more color blood flow; reticulate or branched); (9) calcification (present or absent).

### CEUS image features

The renal angiography phase was divided into either the perfusion phase (0–30 s) or the regression phase (> 30 s). The enhancement and regression mode, peak intensity, enhancement uniformity, enhancement shape, and ring enhancement of the tumor mass were observed. The image features included the following: (1) enhancement mode (earlier enhancement is defined as enhancement of the lesion earlier than the normal renal cortex, slower enhancement is defined as enhancement of the lesion slower than the normal renal cortex, and equal enhancement is equal to the enhancement in the normal renal cortex); (2) regression mode (faster regression is defined as the clearance of intrafocal contrast medium that is faster than that of normal renal cortex, slower regression is defined as the clearance of intrafocal contrast medium that is slower than that of normal renal cortex, and equal regression is defined as the clearance of intrafocal contrast medium is equal to that of normal renal cortex); (3) peak intensity (high enhancement is the enhancement above the normal renal cortex, equal enhancement is equal to the normal renal cortex, and low enhancement is the enhancement below the normal renal cortex); (4) homogeneity of enhancement (homogeneous and heterogeneous lesion enhancements were distinguished based on the uniformity of enhancement intensity distribution); (5) no enhancement (no contrast enhancement was seen in the lesion); (6) the direction of enhancement (centripetal enhancement refers to the enhancement from the periphery to the center of the lesion, and centrifugal enhancement refers to the enhancement from the center to the periphery of the lesion. Diffuse enhancement refers to a simultaneous enhancement of both the periphery and the center of the lesion); (7) the boundary after enhancement (the dividing line between the lesion and the surrounding normal renal cortex is clear and unclear; (8) the range of enhancement (the enhancement area produced by the contrast medium in the lesion site and its surrounding tissue is divided into enlarged and unenlarged); (9) pseudocapsule (whether annular high enhancement around the nodule is observed after the enhancement is divided into pseudocapsule and no pseudocapsule regions).

Evaluation method of early vascular morphology of renal tumor artery using contrast-enhanced ultrasound: The vascular morphology formed through early contrast-enhanced arterial perfusion can be classified as follows: Wheel-shaped type, peripheral vascular type, peripheral nodule type, diffuse type, or unidentified type (unable to distinguish vascular shape and course).

### Statistical analysis

In this study, SPSS v24.0 statistical software was used for statistical analysis. The age and maximum diameter of nodules between the benign and malignant nodule groups were in accordance with normal distribution, and the independent sample t-test was used. Counting data (enhancement mode, regression mode, peak intensity, enhancement uniformity, no enhancement, enhancement direction, post-enhancement boundary, enhancement range, and pseudocapsule) were tested using the χ² test, and the significant difference was set at *P* < 0.05.

## Results

### General situation and ultrasound image features

Table 1 presents the general parameters and results of ultrasound comparison between the benign and malignant groups. There were significant differences in sex, mode of operation, nodule composition, echo, necrosis, and nodule blood flow between the benign and malignant groups (χ² = 10.408, 47.089, 7.481, 20.926, 31.343, and 9.006, respectively; *P* = 0.001, <0.001, 0.003, <0.001, <0.001, and 0.029, respectively). The ultrasound characteristics of malignant nodules were mainly solid (83.85%), hypoechoic (72.7%), and necrosis-accompanied (80.8%); the blood flow in most nodules was between grades II and III (51.5%). The ultrasound characteristics of benign nodules were mainly solid (100%) and hyperechoic (65.9%); the blood flow in benign nodules was between grades 0 and I (73.1%).

**Table 1:** Characteristics of clinical parameters and conventional ultrasound characteristics in the malignant and benign groups

		Malignant (*n* = 99)	Benign (*n* = 41)	χ^2^ or t	*P*-value
Gender, n (%)	Male	61 (61.6%)	13 (31.7%)	10.408	0.001
	Female	38 (38.4%)	28 (68.3%)		
Age (years): mean ± STD		59.290 ± 11.972	55.980 ± 6.984	1.659	0.099
Laterality, n (%)	Left	58 (58.6%)	25 (61.0%)	0.069	0.851
	Right	41 (41.4%)	16 (39.0%)		
Location, n (%)	Superior	34 (34.3%)	9 (22.0%)	2.538	0.281
	Middle	35 (35.4%)	15 (36.6)		
	Inferior	30 (30.3%)	17 (41.5%)		
Surgery, n (%)	Radical nephrectomy	47 (47.5%)	3 (7.3%)	47.089	< 0.001
	Partial nephrectomy	52 (52.5%)	24 (58.5%)		
	Unoperated	0 (0%)	14 (29.3)		
Component, n (%)	Solid	83 (83.85)	41 (100%)	7.481	0.003
	Solid-cystic	16 (16.2%)	0 (0%)		
Echogenicity, (n/%)	Hyper-	25 (25.3%)	27 (65.9%)	20.926	< 0.001
	Iso-	2 (2.0%)	1 (2.4%)		
	Hypo-	72 (72.7%)	13 (31.7%)		
Boundary, n (%)	Well defined	92 (92.9%)	37 (90.2%)	0.289	0.731
	Poorly defined	7 (7.1%)	4 (9.8%)		
Shape, n (%)	Regular	90 (90.9%)	37 (90.2%)	0.015	1.000
	Irregular	9 (9.1%)	4 (9.8%)		
necrosis, n (%)	present	80 (80.8%)	13 (31.7%)	31.343	< 0.001
	absent	19 (19.2%)	28 (68.3%)		
CDFI, n(%)	0	12 (12.1%)	11 (26.8%)	9.006	0.029
	I	36 (36.4%)	19 (46.3%)		
	II	21 (21.2%)	6 (14.6%)		
	III	30 (30.3%)	5 (12.2%)		
Calcification, n(%)	Yes	5 (5.1%)	1 (2.4%)	0.482	0.671
	No	94 (94.9%)	40 (97.6%)		
Tumor diameter(cm): mean ± STD		4.771 ± 2.434	4.283 ± 2.077	1.124	0.263

### Comparison of the characteristics of C-CEUS and H-CEUS in the malignant group

**Fig. 1:**
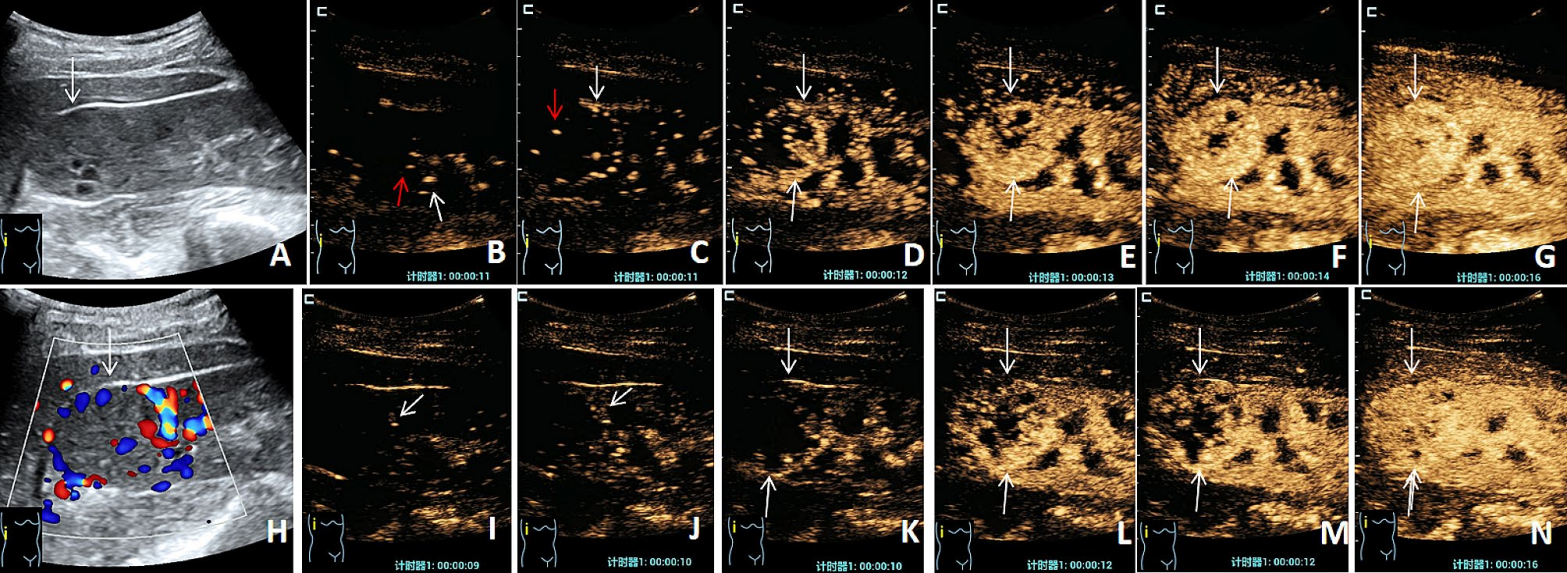
C-CEUS and H-CEUS images of malignant nodules. The nodule was pathologically confirmed as clear cell renal cell carcinoma. (**A**) A shallow echo mass on the superior pole of the right kidney with a clear boundary, regular shape, and anechoic area. (**B**) The time of arrival of C-CEUS, which first arrives at the periphery (white short arrow) and center (red short arrow) of the lesion. (**C-F**) The C-CEUS perfusion process: the contrast medium diffuses enhancement from the periphery and center. (G) The C-CEUS peak time image shows non-uniform high enhancement. (**H**) Rich blood flow signals are in focus, grade III. (**I**) The time of arrival of H-CEUS, which first arrives at the periphery of the lesion (white short arrow). (**J-M**) The perfusion process of the contrast medium: the contrast medium enters from the periphery to the center, showing centripetal enhancement. (**N**) The peak time of H-CEUS shows non-uniform high enhancement (the long white arrow indicates the extent of the lesion)

There were significant differences in the regression mode and enhancement direction of the two CEUS techniques in the malignant group (χ^2^ = 6.782 and 13.771, respectively; *P* = 0.034 and 0.001, respectively). C-CEUS demonstrated mainly diffuse enhancement (49/99, 49.49%), H-CEUS mainly centripetal enhancement (66/99, 66.67%), C-CEUS and H-CEUS both exhibited mainly slow regression (54/99 and 52/99, respectively), but H-CEUS had no isoregressive mode. All ninety-nine lesions could be distinguished from the normal renal cortex on the regression mode. There was no significant difference in enhancement mode, peak intensity, enhancement uniformity, no enhancement, post-enhancement boundary, enhancement range, and pseudocapsule between the two groups (*P* > 0.05) (Fig. 1; Table 2).

**Table 2:** Comparison of features on C-CEUS and H-CEUS in malignant and benign groups*

	malignant(*n* = 99)				benign(*n* = 41)			
	C-CEUS	H-CUES	*χ²*	*p* Value	C-CEUS	H-CUES	*χ²*	*p* Value
Enhancement mode								
Earlier enhancement	68	64	4.003	0.135	5	6	0.158	0.924
Equal enhancement	12	6			8	7		
Slower enhancement	19	29			28	28		
Regression mode								
Faster regression	39	47	6.782	0.034	5	1	2.940	0.230
Equal regression	6	0			3	4		
Slower regression	54	52			33	36		
Peak intensity								
High enhancement	73	67	3.940	0.139	14	10	1.288	0.525
Equal enhancement	7	3			5	4		
Low enhancement	19	29			22	27		
Homogeneity								
Homogeneous	21	19	0.125	0.723	28	15	8.264	0.004
Heterogeneous	78	80			13	26		
No enhancement								
Yes	76	77	0.029	0.865	7	6	0.091	0.762
No	23	22			34	35		
Fill-in direction								
Centripetal	40	66	13.771	0.001	15	22	2.649	0.266
Entirety	49	28			22	15		
Centrifugal	10	5			4	4		
Boundary after enhancement								
Clear	95	96	0.148	0.700	41	40	1.012	0.314
Unclear	4	3			0	1		
Range of enhancement								
Enlarged	4	3	0.148	0.700	0	1	1.012	0.314
Unenlarged	95	96			41	40		
Pseudocapsule								
Yes	82	81	0.035	0.852	9	6	0.734	0.391
No	17	18			32	35		

*Data represent number of nodules

### Comparison of the characteristics of C-CEUS and H-CEUS in the benign group

**Fig. 2:**
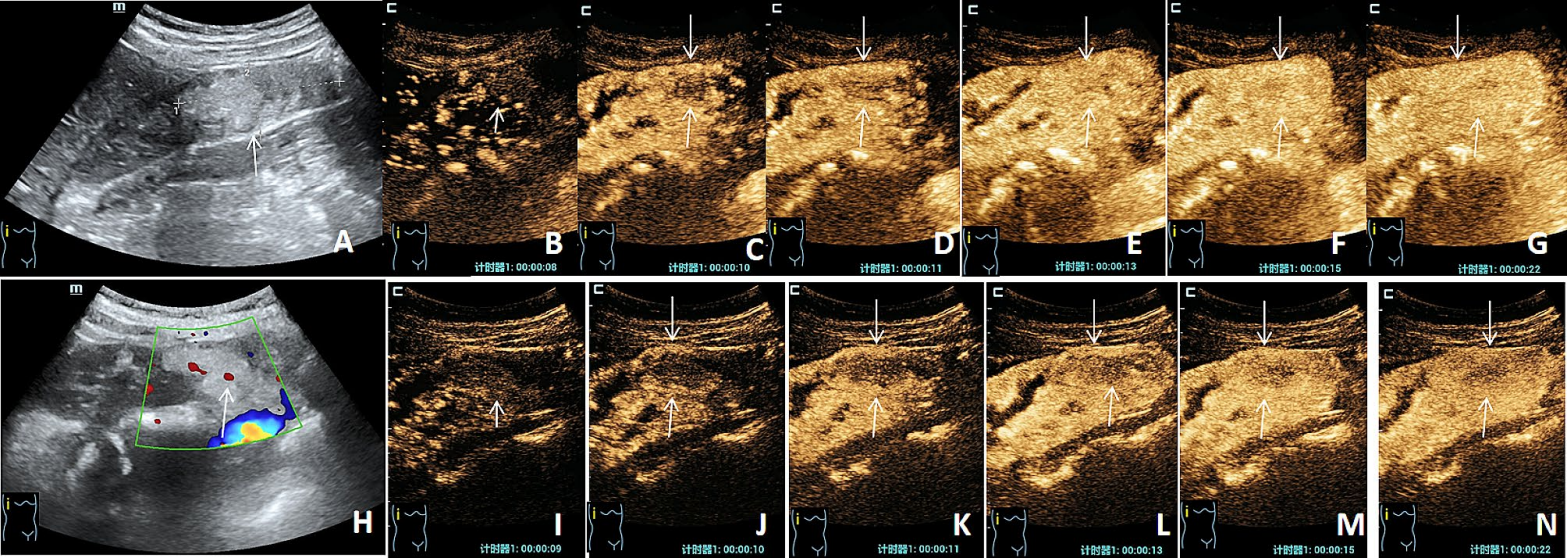
C-CEUS and H-CEUS images of benign nodules. The nodule was pathologically confirmed as AML. (**A**) A hyperechoic mass located at the inferior pole of the right kidney with a clear boundary and regular shape. (**B**) The time of arrival of C-CEUS, which first arrives at the periphery of the lesion (white short arrow). (**C-F**) The perfusion process of the contrast medium: the contrast medium enters from the periphery to the center, showing centripetal enhancement. (**G**) The c-CEUS peak time image shows a slightly uniform low enhancement. (**H**) CDFI showing punctate blood flow signal in focus, grade I. (**I**) The time of arrival of H-CEUS, which first arrives at the periphery of the lesion (white short arrow). (**J-M**) The perfusion process of the contrast medium: the contrast medium enters from the periphery to the center, showing centripetal enhancement. (**N**) The peak time of H-CEUS shows non-uniform low enhancement (the long white arrow indicates the extent of the lesion)

There was a significant difference in enhancement uniformity between C-CEUS and H-CEUS in the benign group (χ ²= 8.264, *P* = 0.004). In 41 cases of benign lesions, the homogeneity of C-CEUS was mainly enhanced (28/41, 68.29%), whereas that of H-CEUS was inhomogeneous (26/41, 63.41%). There was no significant difference in enhancement mode, fading mode, peak intensity, direction, no enhancement, post-enhancement boundary, enhancement range, and pseudocapsule between the two modes (all *P* > 0.05) (Fig. 2; Table 2).

### Comparison of the early enhancement morphologies of C-CEUS and H-CEUS in malignant nodules of varying sizes during the arterial phase

Ninety-nine cases of malignant nodules were divided into ≤ 4 cm group (*n* = 48) and > 4 cm group (*n* = 51) based on the maximum diameter. We then compared the differences in early arterial morphologies between the two groups with varying size nodules under different angiographic modes. The results demonstrated a difference between CEUS and H-CEUS only in displaying early enhancement in the arterial phase in the group of 48 nodules (χ^2^ = 11.421, *P* = 0.022). In the group with a few nodules, 65.58% (31/48) of H-CEUS could show the early vascular morphology of the artery, including 8 cases of spoke vessels, 5 cases of peripheral vessels, 18 cases of peripheral nodules, the other 12 cases showed diffuse enhancement, and only 5 cases could not determine the vascular morphology. In C-CEUS, 31.25% (15 /48) could show the early vascular morphology of the artery, including 3 cases of spoke vessels, 4 cases of peripheral vessels, 8 cases of peripheral nodules, 22 cases of diffuse enhancement, and 11 cases of uncertain early enhancement. In the nodule group with a diameter of > 4 cm, there was no significant difference in the shape of early enhancement in the arterial phase between the two modes (*P* > 0.05) (Table 3).

**Table 3:** Comparison of early enhanced morphology of C-CEUS and H-CEUS in arterial phase of 99 cases of malignant nodules in different sizes*

	≤ 4 cm(*n* = 48)					>4 cm(*n* = 51)				
	Wheel- shaped	Peripheral vessels	Peripheral nodule	Diffuse	Unidentified	Wheel- shaped	Peripheral vessels	Peripheral nodule	Diffuse	Unidentified
C- CEUS	3	4	8	22	11	2	5	10	20	14
H- CUES	8	5	18	12	5	5	4	13	19	10
*χ²*	11.421					2.480				
*P*	0.022					0.648				

*Data represent the number of nodules

## Discussion

The kidney, in contrast to other bodily organs, is small yet abundant in blood supply, with its blood flow constituting a substantial one-fifth to one-fourth of that of the heart. Therefore, the dosage of ultrasound contrast agent should be reduced, and usually, 1–1.5 mL can better show the characteristics of blood perfusion [18]. Excessive dose may result in heightened enhancement intensity during the arterial phase, and the strong echo leads to the nonlinear saturation and artifact interference of video, which not only severely affects the display of pathological details but also causes attenuation because of the excessive concentration of microbubbles in the superficial renal cortex, affecting the imaging and observation of deep renal tissue, resulting in the false appearance of decreased perfusion and corresponding errors in quantitative parameters [19]. Consequently, the contrast dose used in this study was 1.0 mL, and we excluded the patients whose tumor location deeply affects the contrast imaging. The present study exhibited significant differences in nodule composition and operation mode between the malignant and benign groups (*P* < 0.05). The benign nodule group did not contain any solid-cystic nodules, whereas the malignant group accounted for 16.16% (16 /99) of the nodules. Malignant tumors tend to grow faster, prone to bleeding and necrosis, and exhibit mixed cystic nodules. In the benign group, 58.54% (24/41) underwent partial nephrectomy, whereas 7.31% (3/41) underwent total nephrectomy (postoperative pathology was epithelioid AML). All cases in the malignant group underwent surgery. Among the 24 cases of benign renal tumors treated with partial nephrectomy, there were 11 cases of AML (diameter > 5 cm), 8 cases of anadipose AML, 4 cases of oncocytoma, and 1 case of posterior renal adenoma. Therefore, a timely and accurate diagnosis of benign and malignant renal tumors can prevent patients from undergoing unnecessary surgery.

Ultrasound technology provides a fast, safe, repeatable, and cost-effective way to display the basic features of the lesion, such as location, size, shape, boundary, echo, and blood supply. There were significant differences in conventional ultrasound parameters, including an echo of mass, presence of necrosis, and abundant blood flow, in CEUS between the benign and malignant groups (all *P* < 0.05). The benign tumor showed A hyperechoic mass (65.9%), the blood flow was not abundant (grades 0 and I blood flow accounted for 73.1%), and the malignant tumor was mainly hypoechoic (72.7%), and the blood flow was abundant (grades II and III blood flow accounted for 51.5%). Routine ultrasound can distinguish between benign and malignant renal tumors to some extent. However, a previous study [20] reported that 30%∼60% of small RCC exhibited high echo through conventional ultrasound, which was difficult to distinguish from high echo AML, and some malignant tumors lacked the characteristics of high-speed blood flow. Although the blood flow was rich, it was mainly low-speed blood flow. Because of the instrument’s limitations and tumor location, conventional color ultrasound often failed to display low-speed blood flow or deep, small intra-tumor blood flow.

CEUS can clearly and sensitively reflect the blood perfusion state of microcirculation, meaning that on the basis of conventional ultrasound examination, intravenous injection of ultrasound contrast agent can enhance the blood flow scattering signal of the human body, observe the microvascular perfusion information of tissue in real-time, dynamically improve the detection rate of lesions, and distinguish between benign and malignant lesions [21]. According to a previous report [22], CEUS findings of RCC are mostly fast forward and rapid regression with high enhancement, but most renal tumors are rich in blood supply, which does not have a strong diagnostic specificity. Because of the rapid perfusion of C-CEUS to renal lesions, the acquisition of blood flow signals in the arterial phase is limited, and consequently, the disease diagnosis is subjected to some restrictions. H-CEUS is a way to further improve the temporal resolution of the image by increasing the acquisition frame rate and evaluating vascular enhancement with higher temporal and spatial correlation resolution, especially microvascular enhancement [23].

The present study compared two contrast modes in benign and malignant lesions. The results demonstrated a significant difference in the enhancement direction of the two CEUS modes in the malignant group (*P* = 0.034 and 0.001, respectively); C-CEUS showed diffuse enhancement (49/99, 49.49%). However, with the increase in the image frame rate, 66.67% (66/99) of the malignant renal tumors began to enhance from the periphery of the lesion and exhibited centripetal perfusion. This phenomenon is because mainly the increased frame rate can clearly demonstrate that the contrast-enhanced area first appears in the lesion, and the time-varying process of the enhanced area is the direction of contrast medium perfusion. However, in the case of a low image frame rate, the first location of the contrast-enhanced area cannot be precisely depicted; instead, the contrast-enhanced area appears almost simultaneously in all parts of the lesion, so it appears as a whole perfusion. However, no significant difference existed in the enhancement direction of the two imaging modes in the benign nodule group because of the lack of blood vessels in the benign nodule, the slow process of blood perfusion in the nodule, and the perfusion process could be shown using C-CEUS and H-CEUS. Malignant tumors are nodules with rich arterial blood perfusion; consequently, the perfusion process is significantly faster than that of benign tumors. Therefore, H-CEUS can track the fast-moving blood flow in the artery in a complete field of view.

The present study demonstrated significant differences in the regression modes of the two contrast-enhanced ultrasound techniques in the malignant group. Slow regression was dominant in C-CEUS and H-CEUS (54/99 vs. 52/99), with no isoregression in H-CEUS. In 99 cases of malignant lesions, the regression mode of H-CEUS could be distinguished from that of the normal renal cortex, whereas 6 cases of C-CEUS could not be determined, exhibiting the same regression with the surrounding renal cortex. The possible reason is that H-CEUS increases the number of image acquisition, and the frame rate increases from 10 Hz of C-CEUS to 35–45 Hz of H-CEUS, which substantially improves the time resolution and increases the image information. H-CEUS significantly reduces the damage of the contrast medium, thus prolonging its duration in the late stage and making the extinction mode more apparent [24].

The benign group had a statistically significant difference in only enhancement uniformity between the two imaging modes (*P* = 0.004). The C-CEUS of 68.29% (28/41) benign nodules showed enhanced homogeneity, whereas 63.41% (26/41) in the H-CEUS mode showed increased inhomogeneity. The possible reason is that the benign renal tumors often have few microvessels, slow flow rate, difficulty combining with necrosis, homogeneous echo, and uniform perfusion of C-CEUS contrast medium. However, by increasing the frame rate, we can clearly see where the contrast-enhanced area first appears in the interior of the lesion and clearly show the perfusion direction and blood flow volume of the contrast medium in the real case. As time passes, the error of contrast-enhanced ultrasound imaging is reduced [25], and the inhomogeneity of the benign group is enhanced more apparently in the H-CEUS mode. Furthermore, the increase of the frame rate will not cause damage to the contrast medium microbubbles, thus avoiding any potential effect on the diagnostic results [26].

A previous study [27] suggested that the contrast-enhanced ultrasonographic findings of RCC may be affected by mass size. Jun Jiang [28] reported that there was no statistical significance between the peak intensity of CEUS of ccRCC and tumor size, but there was a good correlation between enhanced homogeneity and the frequency of pseudocapsule and tumor size. The frequency of homogeneity enhancement (72%) in tumors with diameters ≤ 3 cm was significantly higher than that in tumors > 3 cm (9%). The present study was divided into groups based on the nodule size and further evaluated the difference between C-CEUS and H-CEUS in displaying early enhancement morphology in the arterial phase in malignant nodules. The results demonstrated a difference in showing early enhancement morphology in the arterial phase between CEUS and H-CEUS in the nodule group (≤ 4 cm) (*P* = 0.022). In the group of small nodules, 64.58% (31 /48) in the H-CEUS mode could show the early vascular morphology of the artery, including 8 cases of spoke vessels, 5 cases of peripheral vessels, 18 cases of peripheral nodules, 12 cases showed diffuse enhancement, and only 5 cases could not determine the vascular morphology. In the C-CEUS mode, only 31.25% (15/48) could show the early vascular morphology of the artery, including 3 cases of spoke vessels, 4 cases of peripheral vessels, 8 cases of peripheral nodules, 22 cases of diffuse enhancement, and 11 cases of uncertain early enhancement. However, there was no significant difference in the shape of early enhancement in the arterial phase between the two angiography modes in nodules with diameters > 4 cm. The reason may be that the frame frequency of C-CEUS does not meet the requirements of accurately displaying small nodules to enhance vascular morphology. The characteristics of small lesions are not as typical as those of large lesions. Furthermore, Yang et al. [29] considered that the size of 4 cm was the dividing point of RCC, and there were differences in the degree of compression of blood vessels, adjacent normal cortex, and the number of arteriovenous fistulas between RCC lesions (> 4 cm vs. ≤4 cm). Microbubbles—the main component of the CEUS contrast agent—cannot spread to the extracellular space. However, their strong scattering characteristics enhance the echo intensity of blood, thereby displaying the morphological structure of blood vessels through the path of contrast agent microbubbles in blood vessels, showing tiny capillaries and improving the sensitivity of blood flow detection [29]. Coupled with the high frame frequency characteristics of H-CEUS, the time resolution is enhanced by improving the frame rate. By capturing fast-moving microbubbles more effectively, it is possible to obtain more contrast medium microbubble information, which enables a clearer display of the microvessels [30]. Therefore, in the present study, the difference between H-CEUS and C-CEUS is statistically significant when comparing early enhanced morphology in the arterial phase of malignant nodules (≤ 4 cm), which represents the advantage of H-CEUS to improve the visualization of tumor microvessels.

There are some limitations in this study: (1) the sample size of patients is small, only benign and malignant renal tumors are discussed, and different pathological types of RCC are not grouped; (2) the clinical value of H-CEUS in the differential diagnosis of the renal tumor must be further evaluated;(3) Solid-cystic benign tumors were not included in this study, which limits the applicability of the study results. The next study will include such patients for further investigation.

## Conclusions

Increasing the CEUS frame rate can improve the time resolution of contrast-enhanced ultrasound images of renal space-occupying lesions. Compared with C-CEUS, H-CEUS is more capable of displaying real changes in the arterial phase and regression phase. In RCC with maximum diameters ≤ 4.0 cm, H-CEUS can help show the perfusion pattern and vascular morphology in the arterial phase, which can make up for the deficiency of CEUs.

## Data Availability

Data is available upon reasonable request.

## References

[1] Barr RG, Peterson C, Hindi A (2014). Evaluation of indeterminate renal masses with contrast-enhanced US: a diagnostic performance study. Radiology.

[2] Kazmierski B, Deurdulian C, Tchelepi H (2018). Applications of contrast-enhanced ultrasound in the kidney. Abdom Radiol (NY).

[3] Bahadoram S, Davoodi M, Hassanzadeh S (2022). Renal cell carcinoma: an overview of the epidemiology, diagnosis, and treatment. G Ital Nefrol.

[4] Winters BR, Gore JL, Holt SK (2015). Cystic renal cell carcinoma carries an excellent prognosis regardless of tumor size. Urol Oncol.

[5] Pavlakis GM, Sakorafas GH, Anagnostopoulos GK (2004). Intestinal metastases from renal cell carcinoma: a rare cause of intestinal obstruction and bleeding. Mt Sinai J Med.

[6] Olson MC, Abel EJ, Mankowski Gettle L (2019). Contrast-enhanced Ultrasound in renal imaging and intervention. Curr Urol Rep.

[7] Zeng SE, Du MY, Yu Y (2022). Ultrasound, CT, and MR Imaging for Evaluation of Cystic Renal Masses. J Ultrasound Med.

[8] Roussel E, Campi R, Amparore D, On Behalf Of The European Association Of Urology Eau Young Academic Urologists Yau Renal Cancer Working Group (2022). Expanding the role of Ultrasound for the characterization of renal masses. J Clin Med.

[9] Selby NM, Williams JP, Phillips BE (2021). Application of dynamic contrast enhanced ultrasound in the assessment of kidney diseases. Curr Opin Nephrol Hypertens.

[10] King KG (2020). Use of contrast Ultrasound for Renal Mass evaluation. Radiol Clin North Am.

[11] Aggarwal A, Goswami S, Das CJ (2022). Contrast-enhanced ultrasound of the kidneys: principles and potential applications. Abdom Radiol (NY).

[12] Rübenthaler J, Bogner F, Reiser M (2016). Contrast-enhanced Ultrasound (CEUS) of the kidneys by using the Bosniak classification. Ultraschall Med.

[13] Reimann R, Rübenthaler J, Hristova P (2015). Characterization of histological subtypes of clear cell renal cell carcinoma using contrast-enhanced ultrasound (CEUS). Clin Hemorheol Microcirc.

[14] Rübenthaler J, Reimann R, Hristova P (2015). Parametric imaging of clear cell and papillary renal cell carcinoma using contrast-enhanced ultrasound (CEUS). Clin Hemorheol Microcirc.

[15] Liu H et al. Cao Hongli,Chen LinThe quantitative evaluation of contrast-enhanced ultrasound in the differentiation of small renal cell carcinoma subtypes and angiomyolipoma.[J].Quant Imaging Med Surg, 2022;12(1): 106–118. 10.21037/qims-21-248 PMC8666748 34993064

[16] Nicolau C, Antunes N, Paño B, Sebastia C (2021). Imaging characterization of renal masses. Med (Kaunas).

[17] Giangregorio F, Garolfi M, Mosconi E (2023). High frame-rate contrast enhanced ultrasound (HIFR-CEUS) in the characterization of small hepatic lesions in cirrhotic patients[. J] J Ultrasound.

[18] Cannella R, Pilato G, Mazzola M (2023). New microvascular ultrasound techniques: abdominal applications. Radiol Med.

[19] Barr RG (2022). Use of lumason/sonovue in contrast-enhanced ultrasound of the kidney for characterization of renal masses-a meta-analysis. Abdom Radiol (NY).

[20] Oh TH, Lee YH, Seo IY (2014). Diagnostic efficacy of contrast-enhanced ultrasound for small renal masses. Korean J Urol.

[21] Squires JH, Fetzer DT, Dillman JR (2022). Practical contrast enhanced liver ultrasound. Radiol Clin North Am.

[22] Houtzager S, Wijkstra H, de la Rosette JJ (2013). Evaluation of renal masses with contrast-enhanced ultrasound. Curr Urol Rep.

[23] Fei Xiang,Han Peng,Jiang Bo (2022). High Frame rate contrast-enhanced Ultrasound helps Differentiate Malignant and Benign Focal Liver lesions.[J]. J Clin Transl Hepatol.

[24] Huang CZW,Gong, Ping et al. Super-resolution ultrasound localization microscopy based on a high frame-rate clinical ultrasound scanner: an in-human feasibility study.[J].Phys Med Biol, 2021;66(8):10.1088/1361-6560/abef45. 10.1088/1361-6560/abef45 PMC8486312 33725687

[25] Lianhua FXLN et al. Value of high frame rate contrast-enhanced ultrasound in distinguishing gallbladder adenoma from cholesterol polyp lesion.[J].Eur Radiol, 2021;31(9): 6717–6725. 10.1007/s00330-021-07730-2 33569621

[26] van Helvert Majorie,Engelhard Stefan,Voorneveld Jason, et al. High-frame-rate contrast-enhanced ultrasound particle image velocimetry in patients with a stented superficial femoral artery: a feasibility study.[J].Eur Radiol Exp, 2022;6(1): 32. 10.1186/s41747-022-00278-w PMC9256892 35790584

[27] Zhu J, Li N, Zhao P (2023). Contrast-enhanced ultrasound (CEUS) of benign and malignant renal tumors: distinguishing CEUS features differ with tumor size. Cancer Med.

[28] Jiang J, Chen Y, Zhou Y (2010). Clear cell renal cell carcinoma: contrast-enhanced ultrasound features relation to tumor size. Eur J Radiol.

[29] Yang LYK, ,Jincun Z (2021). Characteristics of contrast-enhanced ultrasound for diagnosis of solid clear cell renal cell carcinomas ≤ 4 cm: a meta-analysis. [J]. Cancer Med.

[30] Vos HJ, Voorneveld JD, Groot Jebbink E (2020). Contrast-enhanced high-frame-rate Ultrasound Imaging of Flow patterns in Cardiac Chambers and Deep vessels. Ultrasound Med Biol.

